# Doctors Behaving Badly: Professional Regulation and the Tilt Effect(s) of Public Protection Appeals

**DOI:** 10.1093/ojls/gqaf008

**Published:** 2025-04-08

**Authors:** Paula Case

**Keywords:** professional regulation, doctors, public interest appeals, non-deference, regulatory bargain, professional redemption

## Abstract

Regulation in healthcare has often been accused of protecting the professions and neglecting patients. ‘Public protection appeals’, used to challenge fitness to practise decisions considered to be ‘insufficient’ for the ‘protection of the public’, have created a welcome space for judicial scrutiny. Focusing on doctors, the present study of public protection appeals examines the contours of that scrutiny. It frames these appeals as a recalibration of the metaphorical ‘regulatory bargain’, finding that many of the resulting judgments signal a departure from traditional postures of ‘deference’ in professional regulation jurisprudence and a steady judicial assertion of jurisdiction over the core issue of ‘seriousness’ in doctor misconduct. Further exploration of that heightened scrutiny identifies several strands of new doctrine which fortify the regulatory regime in a variety of directions. This exploration also, however, isolates and critiques the emergence of a ‘*Bolton* gloss’—a seam of cases which tilt decision making towards censure and risk disrupting regulatory strategies which have cultivated a commitment to rehabilitative approaches in the disciplinary process.

## 1. Introduction

Healthcare practitioners are subject to complex regulatory arrangements designed to ensure their ‘fitness to practise’. Regulators investigate and adjudicate an incredibly broad spectrum of allegations giving rise to fitness to practise concerns. That spectrum encompasses: patient safety concerns (from mundane clinical failings^1^ to sensationalised cases, such as the surgeon tattooing a patient’s liver at the close of surgery^2^), demonstrations of a lack of integrity (eg misappropriating prescription forms^3^ or ‘CV dishonesty’^4^), criminal convictions for offences which are far removed from patient care (eg domestic violence,^5^ engaging with child pornography,^6^ unlawful environmental protest^7^ or fare-dodging on public transport^8^) and sexual misconduct (ranging from sexual comments or behaviour towards colleagues^9^ to engaging in sexual relationships with vulnerable patients^10^).

When engaged in policing such fitness to practise concerns, regulators must act in pursuit of ‘protection of the public’—an overarching statutory function which now permeates healthcare professions regulation. Positioned at the helm of the regulatory framework for doctors,^11^ it defines the mandate of the profession’s regulator (in the UK, the General Medical Council, GMC) and has been gradually spliced into many facets of its regulatory operations.^12^ The public protection objective now guides the hand of every other healthcare professions regulator,^13^ including the Professional Standards Authority (PSA), the ‘meta-regulator’ in the healthcare professions domain.^14^ The language of this overarching aim also frames judicial involvement in healthcare professions regulation via the ‘public protection appeal’—a novel addition to the regulatory toolbox and the subject of this article. Public protection appeals (also referred to here as ‘regulatory appeals’) entail a regulator appellant inviting the courts to overturn the first instance decision of a regulator’s ‘fitness to practise panel’ or tribunal in a case of misconduct or deficient performance, where that decision is not ‘sufficient’ for the ‘protection of the public’.^15^

The resulting case law has not, thus far, attracted close academic inquiry,^16^ yet judicial scrutiny via these appeals has the potential to perform a dynamic normative and constitutive role, not least by injecting authoritative meaning into the operationalisation of ‘protecting the public’. This review catalogues and evaluates the niche jurisprudence emerging from public protection appeals, using a lens of deference to examine their impact in moderating the terms of the metaphorical regulatory bargain between the state and the profession. Whilst these appeals are embedded in the regulatory frameworks for all healthcare professions, this article focuses on appeals relating to just one statutory framework, as there are significant variations in how fitness to practise is organised across the professions.^17^ The decision to focus on the framework for doctors is an acknowledgement that, by virtue of the duplicate power of appeal bestowed on the GMC, it is the medical profession that has seen the highest number of appeals.

The second section of this article (‘preliminaries’) examines the role of ‘regulator appellants’ (the GMC and PSA) in bringing these appeals, contextualising the oddness of the GMC being in possession of a power to appeal its own tribunal’s decisions.^18^ A two-pronged analysis follows. Using a dataset of public protection judgments from 2017 to 2024 (outlined in the methodology section (section 3)), section 4 adopts a lens of ‘deference’ to examine the contours of judicial interventions via regulatory appeals. Despite a legacy of judicial deference to regulatory tribunals/panels’ decision making on the seriousness of misconduct, close reading of these cases reveals a divergence of approaches, with at least ‘two styles of judging’ being apparent. One of these firmly positions the tribunal as best placed to make regulatory decisions, whereas the other tends towards judicial activism, with the courts steadily asserting jurisdiction over the core issue of the ‘seriousness’ of the doctor’s conduct. Section 5 then draws out ways in which the markedly less deferential judgments ‘tilt’ fitness to practise decision making in the name of public protection, including by generating doctrine where there was previously discretion, and further consolidating shifts away from a model of self-regulation in which the state (including the courts) would rarely interfere with the regulator’s decisions. It also highlights the emergence of a ‘*Bolton* gloss’, a series of cases relying heavily on solicitors’ regulation jurisprudence and emphasising censure^19^, which may constitute a disruptive force to a general theme of redemption in this area of professional discipline. The fine print of imminent reforms to professional regulation threatens both forms of the public protection appeal, so this is a good time to evaluate their use.^20^

## 2. Preliminaries: The Regulatory Context of Public Protection Appeals

### A. The Search for ‘Bad Apples’ and the Road to Redemption in Fitness to Practise

As the doctors’ regulator, the GMC’s responsibilities under the Medical Act 1983 include: oversight of medical education for future members of the profession;^21^ maintaining a register of qualified practitioners;^22^ operating a scheme of revalidation of licences (which aims to verify every five years that every doctor is fit to practise);^23^ and publishing guidance on ethical and practical dimensions of professional work.^24^ It investigates,^25^ and sometimes prosecutes,^26^ complaints about a doctor before a fitness to practise hearing at a Medical Practitioners Tribunal (MPT). It is these tribunals that determine the appropriate professional sanction in cases of proven misconduct^27^ (the main sanctions being the imposition of conditions on registration, suspension from practice or erasure from the register^28^). Tribunals are steered in this task by the *Sanctions Guidance*, which includes details of likely mitigating and aggravating factors and lists of factors indicating that erasure ‘may’ be appropriate (including causing serious harm to others, sexual offences, dishonesty, abuse of position/trust, putting their own interests above those of the patient and a persistent lack of insight).^29^

The fitness to practise process can be caricatured as a search for ‘bad apples’.^30^ The initial complaint (which may come from a patient, a patient’s family member, the doctor’s employer, a ‘responsible officer’^31^ or a colleague) functions as the trigger event, alerting the regulator to a potentially problematic registrant. At the hearing, the regulatory gaze is then applied to the registrant’s conduct, including the deeds specified in the allegation, but also extending to their behaviour both before and after the event(s) complained of, to determine whether fitness to practice is currently ‘impaired’.^32^ Studies of fitness to practise decision making and court judgments reveal a largely non-punitive, redemptive approach to fitness to practise regulation.^33^ Using this approach, the tribunal signals that, notwithstanding misconduct, professional redemption will often be possible,^34^ provided that the doctor engages with the regulator,^35^ preferably attends hearings,^36^ demonstrates meaningful attempts to remediate the harm done^37^ and shows insight into the reasons for the misconduct and its impact.^38^ This holistic assessment involves detailed scrutiny of ‘attitudinal issues’,^39^ and is used as a means of determining whether the doctor presents an ongoing risk to patients or, at the very least, presents a risk to patients because they cannot be trusted to comply with regulatory requirements in the future.^40^ This style of regulation, which values the preservation of space and incentives for professional redemption, is not set out in statute, but represents the regulator’s chosen style of regulation.

### B. Rebranding Professional Regulation and Recalibrating the Regulatory Bargain

Entrusting the control of fitness to practise procedures to the GMC is a key component of the ‘regulatory bargain’,^41^ generally theorised as existing between the medical profession and the state.^42^ This ‘so called’^43^ ‘notional’^44^ bargain has been an enduring metaphor in healthcare professions regulation and has served as a ‘useful fiction’.^45^ It is engaged to explain (amongst other things) a history of self-regulation in medicine, crystallised in the Medical Act 1858. The Act consolidated the state’s delegation of the regulation of professional conduct to the GMC,^46^ a body which was initially controlled by members drawn from the profession it was trusted to regulate.^47^ The GMC was considered best placed to make decisions about entry to the profession’s register, the policing of fitness to practise and how to sanction registrants who failed to meet required professional standards.^48^ Under this arrangement, the regulator shaped and controlled definitions of acceptable behaviour.^49^ The state would rarely mandate how these tasks were organised, provided the regulator ‘retain[ed] sufficiently tight control over their members’.^50^

The terms of that bargain have been modified over time, with the ‘light touch, minimal state model of professional regulation’ envisaged in the 1858 legislation steadily giving way to a more ‘surveillance heavy interventionist state model’.^51^ Key moments in ‘renegotiation’^52^ and transitioning away from self-regulation have included: (i) changes in the regulator’s constitution from a model of professional majority to one of ‘parity’ between medical and lay membership,^53^ including in the tribunals themselves;^54^ (ii) the ‘decentring’ of regulation^55^ in various guises (including fragmentation of the task of regulating doctors and distributing it across multiple state actors, but also across non-state actors, for example, by incorporating oversight of fitness to practise by employer organisations via the revalidation programme);^56^ and (iii) layers of meta-regulation calling the GMC to account for its fitness to practise decision making.^57^ The collective impact of these changes has been variously described as marking the ‘demise’ of self-regulation,^58^ the introduction of ‘regulated self-regulation’^59^ (or, more recently, ‘shared regulation’)^60^ and Quick’s more cautious sounding of ‘the end of “pure” self-regulation’.^61^

Despite the broad reach of these changes, the author shares Quick’s reticence to confirm the death of self-regulation, it being apparent that relics of self-regulation remain. For example, the GMC ‘self-regulates’—it remains sole author of the *Sanctions Guidance* administered by (its own) tribunals^62^ and largely continues to determine the content of the rules which it administers.^63^ Equally significant is that members of the medical profession retain a substantial (if no longer statistically dominant) role in fitness to practise determinations. Each tribunal includes at least one ‘medical’ member, and some will feature a medical majority of 2:1.^64^ The movement away from self-regulation is a process which has not yet reached its endpoint.

High-profile medical scandals, often framed as betrayals of the regulatory bargain, have driven reform.^65^ The steady infusion of public protection into statutory rhetoric follows this pattern, with public inquiries into healthcare failings (and, sometimes, atrocities), rhythmically banging the drum for healthcare regulation to tilt towards public protection, and specifically patient safety.^66^ The Bristol inquiry into mortality rates at a specialist paediatric heart unit reported in 2001 that professional regulation was not working in patients’ best interests, and it proposed the creation of an oversight body^67^ (now realised as the aforementioned PSA).^68^ Within months, an inquiry into the early demise of many of Harold Shipman’s patients and the negligible contribution that the medical profession and its regulator had made in bringing his crimes to light concluded that regulators had placed a higher premium on the fair treatment of the doctors than on the protection of patients.^69^ Nearly 20 years on, the Paterson Inquiry, reflecting on the legacy of a breast surgeon who left hundreds of patients injured and traumatised after luring them into unnecessary procedures, concluded that current regulatory systems had failed to deliver public protection. New regulators were not the answer, but the current system needed to move to ‘serve patient safety as the top priority’.^70^

The public protection objective represents a distancing of regulators from these past allegations of self-interest^71^ and neglect of patients’ interests. As slogans go, it is preferable to the ubiquitous ‘putting [patients/clients/sea lions] at the heart of everything we do’. Its definition is multi-layered, having been expanded by statute into a ‘tripartite’^72^ formulation which includes the protection and furtherance of: (i) the ‘health and safety of the public’; but also (ii) ‘public confidence in the profession’ (sometimes referred to as ‘the wider public interest’^73^); and (iii) ‘proper professional standards and conduct for the profession’.^74^ Whilst protecting patient safety is surely implicit in protecting the public’s health and safety, it is lamentable that it is neither explicitly named within the tripartite formulation nor specifically prioritised.

Whatever its shortcomings, the wording of the overarching function has become regulatory mantra. Its extended form is repeated throughout fitness to practise guidance,^75^ and this, together with its ritual recital in tribunal decisions,^76^ can be regarded as having a performative^77^ significance or ‘social force’.^78^ This significance potentially has external and internal illocutionary effects. The routine framing of regulatory decisions as driven by ‘public protection’ considerations reiterates and reinforces the terms of the regulatory bargain, with regulators cast as protectors of the public and not the profession. Ritual recital also further internalises ‘public protection’ as the collective rationality of regulatory actors.

Whilst the overarching objective serves as a reminder to regulators of their obligations under the metaphorical bargain, the public protection appeal offers courts the opportunity to take on a role in constituting how public protection is to be achieved. In this sense, the introduction of the public protection appeals jurisdiction can be understood as ‘tilting’ the bargain, by qualifying the regulator’s power to define how professional regulation should meet the needs of public protection. Prior to public protection appeals, judicial involvement with professional regulation was almost exclusively via practitioner appeals,^79^ characterised by doctors arguing for a softening of sanctions in their case. Some counterbalancing was possible through patients bringing judicial review applications to challenge disciplinary outcomes, although these are rare and difficult.^80^ Public protection appeals were explicitly designed as a safeguard against the excesses of self-regulation,^81^ and provide a means for inviting the courts to review regulatory complacency or over-identification with doctors’ interests. The reach of these judgments extends beyond the instant case, forming part of an active feedback loop which, in turn, shapes future regulatory decisions.^82^ The appeal mechanism represents an incremental step on the path away from self-regulation, with judges ultimately given the opportunity to shape fitness to practise. The potency of that appeal is diluted, however, if the courts, when invited to intervene, retreat into traditional/well-worn narratives of deference.

### C. Public Protection Appeals: Sharing the Stage

As with the GMC, the PSA shares the overarching objective,^83^ and its ‘section 29 appeal’ power enables it to challenge fitness to practise decisions (across any of the healthcare professions) where it regards the decision as ‘not sufficient (whether as to a finding or a penalty or both) for the protection of the public’.^84^ Uniquely, in the case of doctors, public protection appeals can be brought not only by the PSA (a ‘section 29 appeal’), but also separately or concurrently by the GMC (a ‘section 40A appeal’^85^).

Some context as to how the GMC acquired a duplicate appeal power against its own tribunal’s decisions may be useful. Designed to counter allegations that the GMC’s historical role as investigator *and* adjudicator violated ‘fair hearing’ requirements,^86^ the Medical Practitioner Tribunal Service (MPTS) was created as a separate arm of the GMC in 2012, being tasked with adjudication of fitness to practise cases which reach a hearing. The separateness of the MPTS from the GMC was further underlined by placing the MPTS on a statutory footing^87^ (albeit still as part of the GMC^88^). In 2015, section 40A was inserted into the Medical Act 1983, granting the GMC a ‘power’^89^ to appeal tribunal decisions, mirroring the current wording of the PSA’s section 29 power.^90^

Public protection appeals are situated in a domain of decision making (professional discipline) bounded by the regulatory bargain’s commitment to non-interference. Judicial scrutiny of fitness to practise decision making was traditionally steeped in the vocabulary of deference, particularly on the core issue of the ‘seriousness’ of the doctor’s misconduct. Judicial convention was to assert that the designated panels and committees were the ‘best possible people’,^91^ and the ‘best equipped’,^92^ to determine questions as to seriousness ‘in light of [their] expertise’,^93^ with reminders that ‘respect must be accorded to the professional judgement of the [tribunal]’,^94^ and that courts should be ‘slow to interfere’ on this ground.^95^ Gauging the seriousness of misconduct was clearly defined as a matter for the profession or, by proxy, its regulator.^96^ A key part of the puzzle of understanding what these appeals have brought to the regulatory bargain lies, therefore, in understanding how far, if at all, the courts have moved on this position. In the two-pronged analysis that follows, this study first explores whether a legacy of deference has constrained judicial input on the issue of public protection. It observes that many of the judgments signal a departure from traditional postures of ‘deference’, and represent a steady assertion of jurisdiction over the core issue of ‘seriousness’ in doctor misconduct. Later, it examines precisely how this judicial involvement has tilted fitness to practise decision making under the banner of public protection.

## 3. Methods and Methodology

This article uses ‘deference’ as an analytical lens to examine 40 regulatory appeal judgments concerning doctors. The use of ‘deference’ here is distinct from that regularly deployed in critical studies of medical law (epitomised by ‘doctor knows best’^97^), and is more aptly summarised as ‘regulator knows best’, a position consistent with the traditional model of self-regulation and its core assumption that the regulator (albeit no longer structurally dominated by the profession, but still substantially shaped by the profession) is best placed to make judgment calls about what constitutes unacceptable conduct and when continued practice poses a risk to the public. The task of tracing ‘deference’ to regulators through appeal decisions is further complicated here by the aforementioned emergence of a separate regulatory ‘arm’ responsible for adjudication (the MPTS). A court judgment which is critical of a regulatory sanction might, in some instances, be regarded as ‘non-deferential’ in its robust challenge of the regulatory decision but simultaneously ‘deferential’ in its agreement with the other regulatory arm of the GMC bringing the appeal. This article traces judicial deference to the original decision maker, that is, the regulator’s adjudicatory body, the MPTS. This is because the primary purpose of the analysis is not to measure levels of deference *per se*, but to investigate deference as part of a broader qualitative assessment of the value of the public protection appeals jurisdiction. Adherence to a legacy of deference to the original decision maker would substantially impede advancement of public protection jurisprudence.

The set of cases examined in this article comprises all reported reasoned^98^ judgments in both the GMC’s ‘section 40A appeals’ and the PSA’s ‘section 29 appeals’ (where they concern doctors) from 2017 to 2024 inclusive. The dataset (reproduced in the Appendix) comprises 40 judgments, and was constructed by creating a combined dataset of appeals from the GMC and PSA lists,^99^ and using the Westlaw UK, LexisNexis UK and Bailii (British and Irish Legal Information Institute) platforms to retrieve them. The start date of 2017 was chosen because it marks the GMC’s first use of its power in the crucial case of *Jagjivan*, a case which also outlined the guiding principles for these appeals.^100^ Where relevant, follow-up action by the tribunals was traced via the GMC’s online register.^101^ Most, but not all of the judgments relate to ‘shared’ appeals, with the GMC and PSA litigating as co-appellants. Cases featuring allegations of dishonesty and/or sexual misconduct dominate the case profile,^102^ with only six of the 40 cases featuring neither of these.

Setting out to track deference to fitness to practice decision makers across public protection appeals poses the question: what does a ‘deferential’ regulatory appeal judgment look like? Although a common focal point in studies of judicial decision making, ‘deference’ has no agreed definition,^103^ nor is there consensus on what its counterpoint might be described as (eg ‘judicial activism’,^104^ ‘non-deferential’,^105^ ‘hard look review’^106^?). Nevertheless, it is possible to contrast more deferential judgments with examples of less deferential review by drawing on the range of largely qualitative^107^ markers identified below. However, as will become clear in section 4, none of these criteria alone, where present, would necessarily justify a conclusion that a judgment could be classed as ‘deferential’.

Each of the 40 cases was examined by reference to the following four markers: (i) judicial rhetoric or *framing* of the decision, primarily what is said on the touchstone issue of ‘appropriate’ levels of deference to the original decision; (ii) ‘*outcomes*’ (ie a quantitative measure of whether the original decision was quashed or upheld, but also, if quashed, whether the court substituted its own findings or sanction); (iii) how stringently the ‘burden of justification’ was *policed*,^108^ in particular, how the court articulated the level of specificity required in the tribunal’s reasoning; and (iv) the extent to which the judgment interfered with an ‘evaluative judgement’ of the tribunal, in particular on the grounds of incorrect balancing or ‘too much weight’ being given to a particular factor.^109^ Under the four markers identified above, less deferential judgments are more likely to demonstrate a number of the following features: (i) minimising the zones of decision making where deference is appropriate; (ii) being more likely to quash the original decision and even substitute its own decision; (iii) requiring high levels of specificity in the tribunal’s reasoning; and (iv) stepping into the realms of interfering with balancing decisions. The four markers structure the discussion that follows, which also, incidentally, serves as a commentary on some of the difficulties of measuring judicial deference.

## 4. Competing Narratives in Public Protection Appeals: Deference Eclipsed or Deference in Sharp Relief?

### A. Intensity of Review and Competing Narratives on Deference

First, the stated threshold for review requires attention, as this can have a significant bearing on the scope the courts have for interfering with tribunal decisions. Confusion over whether section 40A/section 29 cases were properly characterised as a ‘review’ (usually involving a narrower field of challenge based on legality and rationality) or an ‘appeal’ (with a broader remit which can also engage with the proportionality of the original decision and involve the judge substituting their own view of the appropriate outcome on the merits) has permeated the case law. The Court of Appeal in *Sastry* agreed that two doctors, one accused of clinical failings^110^ and the other of sexual misconduct towards a nurse,^111^ had grounds to challenge rejection of their appeals against erasure, as the judge had demonstrated ‘excessive deference’ to the tribunal’s decision.^112^ Public protection appeals, it said, triggered a narrow ‘review’ or ‘supervisory’ jurisdiction, particularly with respect to ‘evaluative judgements’,^113^ and decisions could only be interfered with where there was an ‘error of principle’ or the decision was one which no reasonable tribunal could have reached.^114^ This was contrasted with doctors’ appeals against sanction,^115^ which were ‘unfettered’, attracting a full appellate jurisdiction, with judges required to exercise their own judgment as to whether the sanction was ‘excessive or disproportionate’.^116^

*Sastry*’s logic was not always mirrored in the cases examined here. A number of the cases *do* appear to result in assessments of the proportionality or appropriateness of the sanction, rather than focusing on the narrower question of error or rationality.^117^ This blurring of the threshold complicates attempts to track deference across this set of cases, as different courts may have been applying entirely different thresholds of review. In any event, characterisation of the public protection appeal mechanism as a ‘review’ or otherwise does not necessarily determine the application of a pronounced deferential or non-deferential stance. Scholars of review have noted that thresholds based on narrow rationality/reasonableness grounds or a broader proportionality remit can still sustain either deferential approaches or strong forms of review.^118^

The doctrinal focus in *Sastry* on whether section 40A gave rise to a review or appeal ignores the normative question of whether the standard for review of fitness to practise decisions *should* vary according to the identity of the appellant. Why *should* deference be applied in more generous helpings when the appellant happens to be a proxy for ‘the public’ rather than the doctor? An asymmetry of deference,^119^ preferring the doctor’s appeal over challenges on public protection grounds, does not sit comfortably with the overarching function of public protection. Moreover, *Sastry*’s endorsement of deference in these cases also fails to take into account that the appellant in public protection appeal cases is an expert appellant/authority itself. If one of the reasons for deference is ‘expertise’,^120^ then surely it is of diminished importance when the decision is being challenged by an appellant which also has expertise in the relevant field?^121^

### B. Markers of Deference?: ‘Framing’, ‘Outcomes’, ‘Reasoning’ and ‘Weighing’

#### (i) Framing: competing narratives on ‘appropriateness’ of deference

Most public protection judgments explicitly refer to ‘deference’ or ‘deferring’, signalling a shared understanding that levels of deference are important territory in this domain of decision making. Whilst a consensus that ‘diffidence’ or ‘deference’ *should* indeed be paid to the regulatory tribunal’s decision exists, this is bounded by the qualifier of what is ‘appropriate’ in the circumstances.^122^ Appropriateness occupies a broad ‘spectrum’,^123^ bearing similarities to the ‘rainbow’ of approaches observed in judicial review cases.^124^ The hoard of professional regulation case law, comprising thousands of High Court decisions (with higher court judgments being relatively rare), is a recipe for diverse approaches and unresolved tensions. Indeed, judges have noted a confusing array of terms being in use, from directives that courts should be ‘slow to interfere’ or ‘extremely cautious’ to the tribunal’s judgment being ‘virtually unassailable’.^125^

Close reading reveals competing narratives in the public protection appeal judgments, with at least ‘two styles of judging’ being readily discernible.^126^ One firmly positions the tribunal as best placed to make regulatory decisions, ‘reinforcing’ regulatory decision making rather than ‘supplanting’ it.^127^ The other demonstrates a tendency towards judicial activism, with judges importing new legal principles in the place of discretion,^128^ and asserting jurisdiction over the issues of the seriousness of misconduct. Before the substantive dynamics of this judicial activism are addressed in section 5, two critical exemplars are outlined (both being highly influential in the shaping of later cases). These are the cases of *PSA & GMC v Jagjivan* in 2017^129^ (adopting a relatively non-deferential position) and *GMC v Bawa Garba*,^130^ a later Court of Appeal decision in 2018, leaning towards a more markedly deferential approach.

##### (a) Jagjivan—deference eclipsed?

*GMC v Jagjivan*, the first ever section 40A appeal, attempted to systematise judicial statements on deference—gathering together ‘well-settled’ principles applicable to doctors’ appeals which, ‘as appropriately modified’, could be used in public protection appeals.^131^ The *Jagjivan* principles, referenced in almost all^132^ section 40A cases that followed, marked out zones of deference, including that whilst the court should be slow to interfere with ‘findings of fact’,^133^ it was freer to interfere with ‘inferences of fact’,^134^ which it did in *Jagjivan* itself. The appeal court drew an inference that the doctor’s medical advice to the patient to stimulate herself ‘down there’ *was* ‘sexually motivated’, and that the tribunal’s sanction therefore needed to be revisited.^135^ The tribunal’s approach to whether conduct is serious misconduct or results in impaired fitness to practise and what sanctions were needed to maintain public confidence and proper standards in the profession was to be approached with ‘diffidence’, recognising that the court would not have the ‘professional expertise’ of the tribunal.^136^

This background deference was, however, subject to a carving out of ‘matters such as dishonesty or sexual misconduct’ where the court is ‘likely to feel that it can assess what is needed to protect the public or maintain the reputation of the profession’.^137^ This qualifier is nearly always in play. Although a general backdrop of deference is acknowledged in *Jagjivan*, it is eclipsed by the carving out of the zones of sexual misconduct or dishonesty, categories of misconduct which (as already identified) happen to account for the vast majority of public protection appeals.^138^ Under *Jagjivan*, deference was therefore *not* the order of the day.^139^

##### (b) Bawa-Garba—deference in sharp relief

The position outlined above contrasts with a stance which frames deference as the default position,^140^ this line of authority emanating from an earlier time in the GMC’s history^141^ and lying relatively dormant in public protection appeals until the 2018 Court of Appeal decision in *Bawa-Garba v GMC*.^142^ The *Bawa-Garba* case itself concerned whether a doctor controversially convicted^143^ of gross negligence manslaughter against a backdrop of system failures at the hospital on the day in question should be erased from the register, notwithstanding an assessment that she presented no risk to patient safety. The regulatory appeal against a tribunal decision to suspend rather than erase was initially successful,^144^ but in the Court of Appeal, the judgment’s emphasis was on higher levels of deference being appropriately applied to ‘evaluative judgements’ or ‘multifactorial decisions’, also described as ‘jury type questions, where reasonable people might disagree’.^145^ These terms have been applied to issues of public confidence,^146^ assessments of a doctor’s insight and attitudinal issues,^147^ and determinations on the appropriate sanction.^148^ The judgment drew support from the sexual misconduct case of *Marinovich v General Medical Council*, noting that ‘seriousness of the misconduct is essentially a matter for the [tribunal] in the light of its experience. It is … best qualified to judge what measures are required to maintain the standards and reputation of the profession’.^149^

When read together, *Jagjivan* and *Bawa-Garba* are not entirely incompatible, but apply distinct filters on the determinants of deference. The *Jagjivan* filter emphasises the types of misconduct at stake, whereas key passages of *Bawa-Garba*, which are frequently repeated,^150^ stress the form of the decision being challenged (decisions as to sanction, impairment, public confidence and seriousness being ‘evaluative judgements’ and less amenable to challenge). As most section 40A appeals concern issues of sanction or impairment, this reading of *Bawa-Garba* puts most cases potentially into the heightened deference category, mirroring the conclusion above regarding *Jagjivan*.

The criss-crossing of case law here has been exploited in argumentation, with appellants and respondents framing their case to set the court on a path to either deference or close scrutiny. Therefore, although *Jagjivan* identified what appeared to be some bright line rules around *types* of misconduct, not every case has deployed that classification. In *GMC v Raychaudri*, the issue of whether the doctor was guilty of dishonesty by partially completing an assessment form before seeing the patient was treated as a ‘multifactorial’ decision, a conclusion clearly at odds with *Jagjivan*’s classification and opening up the route to greater deference.^151^ In a similar vein, in the sexual misconduct case of *Ahmed*, the appropriateness of suspension over erasure was treated as an evaluative decision, justifying a profoundly deferential approach,^152^ and in *GMC v Mok*, concerning a rape allegation, *Bawa-Garba* was cited as the leading case on section 40A and the decision being reviewed was characterised as an evaluative one of ‘sanction’.^153^ The fact that the court adopts a framing of the issues as ‘evaluative and multifactorial’ does not *necessarily* indicate a deferential decision.^154^ However, it is notable that in *Raychaudri* (alleged dishonesty in partially completing assessment form before seeing a patient), *Ahmed* and *Mok* the court refused to disturb the tribunal’s decision.^155^ In fact, in all of the ‘unsuccessful’ section 40A appeals post-*Bawa Garba*, the courts reiterated what was said in *Bawa-Garba* in preference to *Jagjivan*.

#### (ii) Using ‘outcomes’ to measure deference

In the majority of cases (29 of the 40 reasoned judgments), the public protection appeal succeeded and the tribunal’s decision was quashed, and in 11 of these the court went further, substituting its own decision, usually erasure.^156^ Whilst the relationship between appeal outcomes and deference is non-linear,^157^ there does seem to be a broad correlation between successful appeals (the court agreeing that the tribunal decision was so flawed that the decision could not stand) and explicit non-deference.^158^

However, a majority of successful appeal outcomes is far from being a cast iron indicator of relative non-deference. A high ‘success’ rate can just as easily be read as a product of the cases selected for review as it can an indicator of relatively high levels of judicial activism. The creation and use of the GMC’s public protection appeal needs to be viewed in its wider political context here. The introduction of section 40A was, as explained earlier, part of building the narrative of separation of the GMC from the MPTS.^159^ Each appeal can be viewed as having its own additional performative significance,^160^ in its positioning of the GMC and MPTS as adversaries in the courtroom, thereby emphasising their separateness and reinforcing the public-facing narrative of institutional independence.^161^ The profile of section 40A cases shows that the GMC has (unsurprisingly) selected cases where a court is most likely to be receptive to challenge and the appeal is most likely to succeed (ie dishonesty and sexual misconduct—domains where the courts have fairly consistently highlighted the court’s right to assert its own competence to determine the issue, notwithstanding the expertise of the tribunal).^162^ Judicial condemnation of dishonesty and lack of sexual probity in the professions has resulted in two lines drawn relatively deep in the jurisprudential sand,^163^ and these represent safe territory for appeals. There will be little resistance from the courts to a norm that findings of sexual misconduct or dishonesty should be met with erasure, no matter the extent of the doctor’s insight or remediation. That is not to say that dishonesty *always* warrants erasure,^164^ or that breaching boundaries with patients *always* merits exclusion from the profession.^165^ But, in seeking (and obtaining) judicial reinforcement of the dishonesty/sexual misconduct bars, the regulator not only underscores its (philosophical) independence from the MPTS, but also takes a low-risk punt at selected cases where the frequently stated conduct norms of integrity and sexual propriety appear not to have been applied. Bringing the appeal and succeeding may be as much about the expression and performance of authority as about the content of the appeal. The predictable strategic preference for the easier-to-win cases also explains why outcomes, even an *overwhelming* success rate in these appeals, cannot necessarily be equated with a non-deferential stance by the court, and the success rate can be symptomatic of the profile of softer target cases the regulators have chosen to appeal.

Also worth noting is that doctors’ appeals, despite the broader scope of review, are rarely successful.^166^ Appellant identity may therefore have a substantial impact on success rates—regulatory appellants are likely to fare better as ‘repeat players’,^167^ as compared with defendant doctors in fitness to practise proceedings. Implicit reputational factors may also predispose courts to regard a regulatory appellant’s arguments as highly credible. Considering the broader implications of this for measuring judicial deference generally, these confounding variables, such as the impact of the identity of the appellant and the appellant’s strategies for selecting which cases to appeal, may mean that the inter-reliability^168^ of independent studies of judicial deference is likely to be low, each jurisdiction being affected by highly specific variables.

#### (iii) Policing the ‘burden of justification’—reasoning requirements as a marker of deference

Then there is the issue of how closely the burden of justification is ‘policed’^169^—in particular, how demanding is the courts’ application of the tribunal’s duty to give reasons^170^? Some contrasting cases illustrate a mix of approaches at play. In *Mehta*, a case of sexually motivated conduct towards a junior colleague, whilst the court acknowledged that the tribunal had made no mention of certain paragraphs of the *Sanctions Guidance*^171^ (including those stressing the seriousness of sexual misconduct), it was clear that it was ‘alive’ to the relevant principles and ‘to the *Sanctions Guidance* generally’.^172^ Likewise, in *Awan*, it was unnecessary for the tribunal to mechanistically reference relevant paragraphs or authorities as if they were ‘a pilot going through the pre-flight checklist’.^173^

Contrasting with *Mehta* and *Awan* are what are referred to here as ‘the departure cases’. In these cases, a decision was characterised by the appeal court as departing from paragraph 109 of the *Sanctions Guidance*, as the tribunal had determined not to erase, *despite* the presence of a number of the paragraph 109 factors indicating that erasure ‘may’ be appropriate.^174^ In the departure cases, the courts applied a high sufficiency of reasoning bar and employed a creative rewriting of the guidance. In *Donadio* (a key departure case), the factors which ‘may’ indicate the appropriateness of erasure included the presence of regulatory breach, which was newly defined as ‘dishonesty’ in the same case.^175^ This departure triggered a duty to provide sufficient case-specific detail of the reasons for the departure, and failure to do so disclosed an ‘error of principle’ enabling the court to intervene.^176^ It is not clear how suspension followed by review of the doctor concerned qualified as a ‘departure’, given that the guidance is a long way from mandating erasure or anything close to a presumption of erasure in cases of dishonesty, and there is a long tradition of not using erasure where the dishonesty is not persistent.^177^ The approach taken in *Donadio* also heavily qualifies the last-resort principle in the *Sanctions Guidance* (not referred to in the judgment), which preserves erasure for cases ‘where this is *the only means* of protecting the public’.^178^

The ‘departure cases’ give increased potency to the paragraph 109 factors by labelling them ‘indicators of erasure’.^179^ This move creates new doctrine, reconceptualising a fairly broad discretion as to when to impose erasure, into a highly structured decision-making space. The same judgments recast decisions *not* to order erasure where a number of ‘indicators of erasure’ were present as ‘departures’ from the guidance which therefore require ‘detailed case specific justification’.^180^ The effect is to elevate the factors to something close to presumptions of erasure by another name,^181^ ironically itself a ‘departure’ from and rewriting of the guidance.

The *Bramhall* decision, which also employed the ‘departure’ approach to support quashing of suspension, went a step further by requiring what might be termed ‘negative reasoning’. Tribunals apply a ‘bottom-up approach’ to find the least restrictive sanction which would satisfy public protection, whilst not being disproportionate in its impact on the practitioner. This approach mandates that once fitness to practise is deemed impaired, decisions on sanction should proceed by considering the least restrictive option first, working up through the sanctions ladder rung by rung (from no sanction, to conditions, to suspension and so on).^182^*Bramhall* required, however, that the record of the tribunal’s reasoning should not stop once the tribunal had found a sanction it believed to satisfy the public protection and be ‘proportionate’. Instead, it should proceed to explain why any other, more severe sanction was regarded as ‘*dis*proportionate’ and *more than* necessary to satisfy the public protection objective.^183^ Failure to include this further ‘negative’ reasoning was an ‘error’ of principle. This seems to be a superfluous demand, as it is hard to see how this additional requirement does anything more than require the tribunal to state the inverse of its reasoning for finding the sanction chosen as sufficient for protection of the public.

#### (iv) Judicial interference with evaluative judgments

Finally, interference with ‘weighing’ is often regarded as a broad indicator of a higher intensity of review (or non-deference).^184^ As per *Bawa-Garba*, a greater degree of deference is usually considered to be appropriate for those ‘evaluative’ judgments made by the tribunal, yet one of the most common reasons given for quashing the tribunal’s decision is that the tribunal gave ‘too much weight to’ mitigating factors when determining the sanction or that elements of public protection had not been properly ‘balanced’.^185^ Some of the further circumstances in which this judicial interference with weighing occurred and the ramifications of these decisions are explored in detail below.

## 5. Judgment Analysis: Fortifying Boundaries, Ratcheting up the Likelihood of Erasure and Re-writing the Sanctions Guidance

Collectively, the 40 public protection judgments reveal broad, often overlapping, themes of critique, many converging on tribunals failing to ‘grapple with’ the ‘seriousness’ of the doctor’s conduct. This trend marks a real step-change from the convention of deference on the matter of ‘seriousness’ in professional regulation noted above, and, whilst strains of this more deferential reasoning remain, they are very much in the minority.^186^ The following section highlights a number of mechanisms through which (in addition to the ‘departure’ doctrine noted above) the courts have reinforced the regulatory regime, by steadily asserting jurisdiction over the gravity of the case and the defendant’s conduct.

### A. Broadening the Reach of ‘Sexual Misconduct’ and ‘Dishonesty’

The labels ‘dishonesty’ and ‘sexual misconduct’ attract a particularly condemnatory regulatory gaze, heightening the risk of erasure. Public protection judgments have been critical in incrementally extending the reach of both of these labels.

#### (i) Expanding the label of ‘sexual’ misconduct

In the section 40A case of *Chandra*, the Court of Appeal employed powerful rhetoric of the public being ‘entitled’ to be treated and examined by doctors whose ‘trustworthiness and sexual integrity is not and never has been seriously in question’, thereby appearing to ratchet up the likelihood of erasure for doctors accused of either sexual misconduct or dishonesty.^187^ Other judgments have highlighted the seriousness of sexual misconduct towards colleagues and emphasised its relevance to public protection.^188^ Tribunals have also been criticised for not interrogating a doctor’s knowledge of a patient’s vulnerability (as this went to culpability)^189^ and not recognising the experience of a recent diagnosis as a form of vulnerability.^190^

Since the judgment in *Haris*,^191^ concerning allegations that ungloved, intimate examinations of a patient were sexually motivated, non-clinically indicated intimate examinations are now close to being presumptively treated as ‘sexual’. The court recommended that the GMC need not charge such cases as ‘sexually motivated’, this being too easily refuted by a doctor’s claim of asexuality or a lack of positive proof of sexual motive. Issues of motive are, of course, examples of *Jagjivan’*s ‘inferences of fact’, and involve highly evaluative judgments. Nevertheless, *Haris* is part of a broader pattern of negating what Lord Woolf might have called a ‘presumption of beneficence’^192^ once accorded to the medical profession. These appeals show the courts ‘correcting’ the tribunal’s assumption of benign motives^193^ and displacing over-optimistic views of the doctor’s insight and remediation.^194^

#### (ii) Reclassifying regulatory breach as dishonesty

A doctor’s failure to comply with restrictions imposed on their practice (eg interim/final conditions or suspension), or resuming practice when not fully registered or practising outside of their registered specialism, is now captured by the label of convenience, ‘regulatory breach’.^195^ Although frequently treated as misconduct, regulatory breach is not mentioned in the *Sanctions Guidance*. However, public protection appeal judgments now redefine the fundamental character of this type of misconduct as dishonesty.

In the most cited of these cases, *GMC v Donadio*,^196^ the consultant radiologist was made subject to conditions of direct supervision due to a pattern of ‘overcalling’ abnormalities, yet had continued to work without additional supervision for a further 10 days. Collins Rice J found the MPT’s decision to suspend rather than erase was ‘wrong’—the misconduct was a ‘knowing breach of regulatory requirements’. Although the tribunal had identified this as dishonest, it was, in truth, dishonesty of the ‘utmost gravity’,^197^ even though it did not easily fall within the forms of dishonesty identified in the *Sanctions Guidance*.^198^ This judgment was therefore a direct challenge of the tribunal’s view of the ‘seriousness’ of the misconduct and reclassified regulatory breach as giving rise to a presumption of dishonesty.^199^ In a complimentary move, the court in *Mmono* observed that dishonesty in the doctor’s responses to the regulator ‘undoubtedly places his dishonesty at the more serious end of the spectrum’.^200^

Disregard for regulatory requirements strikes at the heart of the regulator’s ability to regulate, and these judgments represent a significant step in shoring up protection of the public, by strengthening the regulator’s hand in what might otherwise be viewed as bland ‘technical breaches’. Such breaches were framed as the registrant ‘not being properly subject to the regime’ or as ‘bypassing’ the licensing regime,^201^ pointing to the ‘burden on medical practitioners, as with all professionals, to engage with the regulator’.^202^ Collectively, the cases rewrite the *Sanctions Guidance*, treating regulatory breach as posing a high risk of erasure, or even a ‘new indicator’ of erasure. Attempts to evade the regulatory gaze, even partially or temporarily, are now imbued with intense regulatory significance and the potential for top-end sanctions is increased.

### B. Placing Too Much Weight on Personal Mitigation?

#### (i) Tempering reliance on positive testimonials

A finding that the tribunal had failed to grapple adequately with the gravity of the doctor’s conduct was frequently accompanied by findings that the tribunal’s view on seriousness was obscured by attaching ‘too much weight’ to the doctor’s mitigation.^203^ Positive testimonials from peers, patients and colleagues can be part of the doctor’s mitigation^204^ and may go some way in persuading the panel that the doctor should be allowed to resume practice. Although largely of narrative value, as part of the ‘story’,^205^ they sometimes carry ‘substantial weight’.^206^ This time-honoured practice is deeply problematic. On the one hand, testimonials can offer further clues or corroboration as to a practitioner’s skills, essential character (a key component of regulatory strategy) and the risk their continued practice presents. The usefulness of a doctor and their risk rating are relevant to considering the best outcome for protection of the public.^207^ On the other hand, this practice can be viewed as peer and patient endorsement (eg Dr Chaudhary, who presented 47 positive testimonials from patients and relatives of patients^208^) being given too much potency in an objective decision about whether a doctor is fit to practise.

Also problematic are cases where there is a paucity of testimonials.^209^ Given the culture of providing testimonials in mitigation, their absence may be taken as an indication of *a lack of* competence or good character, but the significance of an absence of testimonials could as easily be due to a doctor’s unpopularity as a result of raising safety concerns or attributable to the doctor having little experience in the workplace, no embedded networks and/or few contacts in the UK. The appeal cases analysed here demonstrate unease with the tribunal’s reliance on testimonials, including challenges for attaching ‘too much weight’ to positive testimonials, when determining sanction.^210^ In a show of heightened scrutiny, the court in *GMC v Somuah-Boateng* regarded itself as well placed to judge the relevant weight to be attached to testimonials, diminishing their significance on the facts of this case as ‘no more than pleas for mercy’.^211^ And in *Nwachuku*, the court regarded the tribunal as falling into error by stressing the mitigating impact of positive testimonials when they did not address the core issue of the doctor’s honesty.^212^ This scrutiny is essential to keep the weight attached to testimonials in check. Peer endorsement raises the spectre of self-interested regulation, with doctors perceived as being inclined to protect one other,^213^ and patients perhaps being too trusting.^214^

#### (ii) Challenging the use of time and space in fitness to practise sanctions

The judgments reveal a pattern of conflict over the values underpinning sanctions. This includes a particular flashpoint on the regulatory space between a ‘12 month suspension followed by review’ and ‘erasure’,^215^ with a number of appeals resulting in the court substituting suspension with its own order of erasure.^216^ The choice between suspension or erasure in these cases involves a balancing of at least two broad incommensurable goods: the benefits of a redemptive approach to professional sanction^217^ and the signalling or censuring power of erasure as a sanction which unequivocally messages the doctor’s expulsion from the profession, in order to preserve public confidence.^218^

The tribunal’s commitment to redemptive strategies was demonstrated in a number of cases where suspension followed by a review was utilised as a ‘wait and see’ space. The space facilitates the gathering of more evidence of a doctor’s character and gives the doctor time to achieve and demonstrate their insight and remediation, thereby demonstrating that they are fit to resume practising—the suspension from practice ensuring that the public is being protected in the meantime.^219^ Suspension with review is understood by MPTs as a contingency plan, retaining the *potential* for erasure/exclusion—resumption of practice only being possible if fitness to practise is proven at a later date.^220^ This aligns with a ‘forgiveness’-oriented sanctioning regime,^221^ and it is the tribunals especially, with their involvement in ‘review’ hearings, that see the extent of a practitioner’s transformation at the end of the suspension period.

This use of time and space to cultivate insight and remediation is consistent with earlier reports that proven dishonesty is far less likely to trigger erasure in the medical profession as compared with the legal profession, due to the emphasis on redemption in the medical model of fitness to practise.^222^ In *Somuah-Boateng* (sexual relationship with a patient recently diagnosed with multiple sclerosis), the court refused to treat suspension followed by review as close in significance to erasure: ‘Public confidence in the profession is, I find, unlikely to be satisfied by a suspension, whether or not followed by a review.’^223^ Again, this judgment tends to minimise the role of remediation in decision making. It insists that gross breaches of the profession’s norms ought to be visibly met with an unambiguous sanction signalling expulsion (erasure), rather than something open-ended, signalling that the erring registrant *may* be permitted to resume practice and make the same errors again (eg suspension).^224^ The tension noted here shows the courts substituting their own judgment on how public confidence concerns which pull towards a censure model of sanctions and the pursuit of a redemptive approach to sanction are to be balanced.

#### (iii) The ‘Bolton gloss’: disrupting the redemptive approach to sanction?

A broadly similar clash of values is at the crux of a concerning trend in these cases, the effect of which is to urge tribunals to apply a ‘*Bolton* gloss’ to decision making on sanction. The relevant extract cited from *Bolton v Law Society*^225^ states that: ‘the reputation of the profession is more important than the fortunes of any individual member’, therefore mitigation is regarded as of little weight,^226^ and this is used to argue that ‘too much weight’ had been attached to the doctor’s mitigation as part of the reasoning for quashing the tribunal’s decision.^227^ The ‘mitigation’ being referred to was frequently the doctor’s demonstrations of insight and attempts at remediation. So, in *Zafar*,^228^ the tribunal imposed the maximum 12-month suspension with review, citing mitigating factors, including significant remorse, contrition and remediation. The section 40A court noted these elements, but referenced *Bolton*, saying ‘such matters only amount to so much’,^229^ and imposing an order for erasure. In *Chandra*, a doctor’s restoration to the register 11 years after erasure (for having a sexual relationship with a patient) was quashed due to having placed ‘too much weight’ on the doctor’s remediation.^230^ In *Patel*, the court interfered with the tribunal’s decision not to sanction a doctor who showed extensive remediation, reflections, shame and regret, in part because too much weight had been placed on remediation,^231^ noting that the tribunal had failed to heed the guidance in *Bolton*.

However, the pursuit of a closer alignment with *Bolton*, including suggestions that tribunal decisions were flawed if they failed to reference it,^232^ is deeply problematic. *Bolton* was written for a different regulatory scheme (regulators and regulatees of the legal profession over 30 years ago). Extracts from the judgment have since been transplanted into the jurisprudence of doctor regulation,^233^ but by repeated endorsement (including in the cases discussed here), its authority has been given a meaning and significance which the detail of the judgment does not support. Whilst *Bolton* urged that mitigation should be given little weight, Lord Bingham MR’s conception of mitigation was narrow, referencing statements of ‘good character’, the impact of suspension on the solicitor’s practice and concerns that the ‘individual’ interests^234^ of the professional should not be prioritised over what was necessary for the protection of the profession’s reputation.^235^ The bounded field of mitigation envisaged in *Bolton* does not extend to the broad ambit of mitigating factors commonly featuring in modern doctors’ fitness to practise hearings. As highlighted above, the pursuit of redemption where possible has evolved as a central focus of doctors’ fitness to practise regulation,^236^ and the first mitigating factors mentioned in the *Sanctions Guidance* are the development of ‘insight’ and attempts to address or ‘remediate’ the misconduct.^237^ This broader conceptualisation of mitigation reaches far beyond protecting the individual doctor’s interests from harsh sanctions, but has central importance in modelling a fair and just regulatory system,^238^ in assessing the extent to which the doctor presents continuing risks to the public^239^ and in encouraging candour^240^ as a default response from healthcare professionals when things go wrong with a patient’s care or complaints are made. However, these strategies are not clearly captured by *Bolton*’s pronouncements on mitigation.

Yet, this enlarged version of what *Bolton* decided has become an accepted staple of professional regulation jurisprudence, without any detailed normative inquiry undertaken as to the fit of its ‘principles’ with modern regulation. The embellishment of *Bolton*’s message aside, there are strong reasons to question this ‘fit’. First, its vocabulary belongs to a different era.^241^ For example, its focus on the profession’s ‘reputation’, although seemingly assumed to be a perfect proxy for maintaining ‘public confidence’ in the overarching objective,^242^ is highly questionable. At the very least, ‘reputation’ feels inward regarding, and not clearly distinct from the profession’s own interests (considered by *Bolton* to be the profession’s ‘most valuable asset’^243^), whereas public confidence can more readily be viewed as service-oriented and aligned to the interests of patients. Further, *Bolton*’s decree that professionals should be capable of being ‘trusted to the ends of the earth’^244^ seems out of step with modern expectations of professions.^245^ Secondly, the emphasis on redemption in medical professions regulation is miles away from the design of legal professions regulation. The statutory concept of ‘impairment’,^246^ which requires the tribunal to focus on the misconduct in the broader context of evidence to date of the doctor’s current fitness to practise, has generated a model which is quite distinct from a reactive, ‘misconduct invites sanction’ approach.^247^ Thirdly, *Bolton* is being used to challenge decisions that opt for suspension rather than erasure, yet, ironically, *Bolton* referred to the choice between suspension and erasure as an evaluative judgment which disciplinary tribunals were best placed to make.^248^

Above and beyond these difficulties, most concerning is that *Bolton* signalled that protecting the profession’s reputation was the ‘most fundamental’ purpose of professional sanction.^249^ Obviously, *Bolton* does not engage with the wording of the overarching function of public protection^250^—a provision designed for healthcare professions and appearing much later than *Bolton*. Section 1(1A) of the Medical Act 1983 is silent on the equivalence of or prioritisation between the three strands of public protection, but it certainly does not indicate that maintaining ‘public confidence’ is the ‘most fundamental purpose of’^251^ sanctions. In this respect alone, *Bolton* constitutes an inappropriate ‘gloss’ to the public protection mandate set out in statute by superimposing a tilt towards public confidence concerns.

In pulling the MPTs away from reliance on mitigating factors, strands of these cases collectively threaten to disrupt a regulatory strategy which places significant value on insight and remediation. In modern regulatory talk, mitigating factors are not just shorthand for ‘the doctor’s interests’ as envisaged in *Bolton*. They play a central role in assessing risk and gauging the sanction needed in order to protect the public. Whilst it is by no means suggested that there should not be clear limits to the possibilities for professional redemption, the trade-off between pursuit of an approach which values reflection, apology, contrition and forgiveness and the pursuit of action which demonstrates censure where public confidence may be at risk is not really engaged with in these judgments. This may be exactly the kind of evaluative judgment where significant deference to the adjudicative body is justified. The mechanisms which make up this redemptive approach merit deeper interrogation and justification, but few judgments explicitly recognise the role of attitudinal issues.^252^ The practice of calling into play a *Bolton* gloss, which makes use of a narrow definition of ‘mitigation’ as a trump card in these cases, is a heavy-handed and ill-fitting response. It fails to acknowledge and evaluate the benefits of a redemptive approach to fitness to practise and neglects to examine the validity of ‘public confidence’ claims.

## 6. Conclusions

The public protection motif both symbolises and embeds a tilting of focus in the regulatory bargain: it is part of a rhetorical disowning of the past and a rebranding of professional regulation as driven by a commitment to public service. Public protection appeals represent a segment of this process, creating a welcome space for judicial involvement in the regulatory process, but that role is substantially diminished if judicial oversight has maintained traditional narratives of deference.

It is, of course, unrealistic to categorise all cases according to a deference/non-deference binary. Judgments fall somewhere along a spectrum. Some carry conflicting markers of deference and non-deference or are positioned in the middle ground, being neither distinctively one thing nor the other.^253^ The analysis presented here suggests that these cases have made major inroads on judicial deference to tribunal decision making. Despite the characterisation of public interest challenges as a (narrower) ‘review’ mechanism rather than an appeal, a backdrop of case law in which pronouncements of deference are deeply embedded, three-quarters (28) of the 40 cases brought resulted in the court overturning the tribunal’s decision. Seventeen of these judgments preferred a tone of vigilance and close scrutiny, and interfered with the tribunal’s evaluative judgments—stretching the boundaries of ‘review’ and appearing to modify the regulatory bargain by marking a departure from the courts’ hands-off approach. In 11 such cases, the court not only quashed the tribunal’s decision, but also substituted its own decision on the appropriate sanction. Given that so many of these cases preferred a vigilant, non-deferential approach, attention turned to how these judgments have transformed decision making in the name of public protection.

It is unsurprising that one broad impact of public protection appeals is to ratchet up the likelihood of high-end sanctions, making erasure more likely in certain cases. What are more surprising are some of the paths taken to this outcome. The courts have provided welcome fortification of the regulatory regime in strategic territories of sexual misconduct and dishonesty. Failures to comply with regulatory conditions or suspension (once tagged as the rather anodyne ‘regulatory breach’) have been reclassified as dishonesty ‘of the utmost gravity’, inviting a presumption of erasure.^254^ The seriousness of sexual misconduct towards colleagues has been escalated, the approach to non-clinically indicated intimate examinations has been recalibrated and the courts have been seen to correct overly benevolent interpretations of doctors’ motives. Ultimately, these developments have challenged the ‘weighing’ judgments of the tribunal. The courts have grasped their role as ultimate arbiter of issues of the weight to be attached to dishonesty^255^ or other forms of misconduct, its ‘importance’ and, consequently, the matter of seriousness. Collectively, these cases strengthen the regulator’s hand and heighten the likelihood of top-end sanctions. However, this article also noted some prominent lines of reasoning, namely, extending *Bolton* beyond its intended parameters, as threatening to disrupt regulatory strategies which promote redemption. Research is needed into the public protection benefits of a rehabilitative approach to fitness to practise before tribunals and courts further extend and embed the ‘*Bolton* gloss’ to the public protection objective.

All of this is written at a time when the public protection appeal is in peril. The section 40A appeal power will likely simply vanish^256^ once new regulatory tweaks are eventually rolled out to doctors.^257^ Meanwhile, the PSA’s appeal power is likely to be impacted by a (potentially) substantial move away from public fitness to practise hearings to ‘accepted outcome’ decisions made by two case examiners, and reached by consensus with the registrant^258^ ‘behind closed doors’.^259^ Tribunals are under a duty to make a call on the ‘seriousness’ of the doctor’s misconduct, the ‘context’ and the doctor’s ‘character’. This is recognised as being extremely difficult in cases where the doctor has accepted the allegations as presented.^260^ According to *Lingam*, tribunals have a duty to engage with the facts, and, in cases where much had been conceded but there were still be gaps in the narrative (eg here, around the doctor’s risky prescribing practices), the public protection imperative requires tribunals to be ‘bold’ and use questioning to clarify the central issues.^261^*Lingam* raises questions of how a scheme of ‘accepted outcomes’ can deliver on public protection, as this system involves the doctor accepting the facts as presented, *completely* depriving the tribunal service of the opportunity to interrogate the facts. Further analysis of these developments is outside the scope of this article,^262^ but the planned reforms add weight to the impetus to interrogate the impact of these appeals on doctor regulation.

## Footnotes

1
*GMC v Sledzik* [2019] EWHC 189.

2
*PSA & GMC v Bramhall* [2021] EWHC 2109.

3
*Bakare v GMC* [2021] EWHC 3278.

4
*Lateef v GMC* [2022] EWHC 2743.

5
*Ibrahim v GMC* [2022] EWHC 2936.

6 H Khalid, T David and S Ellson, ‘Extreme Medical Misconduct: 49 UK-Registered Doctors Involved in Child Pornography, Dealt with by the General Medical Council 2012–2020’ (2023) 91(3) Medico Legal Journal 148.

7 ‘British Doctor Suspended over Involvement in Just Stop Oil Protests’ *The Independent* (24 April 2024) <www.independent.co.uk/climate-change/news/climate-protest-uk-doctor-suspended-b2533788.html> (unless otherwise stated, all references to URLs were last accessed 31 October 2024).

8 Dr Ip, ‘Great Ormond Street Doctor Suspended for “Serious” Dishonesty’ (*BBC News*, 23 September 2023) <www.bbc.co.uk/news/uk-england-london-65052322>.

9
*Higgins v GMC* [2024] EWHC 1906.

10
*Metastasio v GMC* [2023] EWHC 1918.

11 Medical Act 1983, s 1(1A).

12 As detailed in the GMC’s *Fitness to Practise Rules 2004*; *The Impact of Our Work* (GMC 2018).

13 See eg Dentists Act 1984, s 1(1ZA); Opticians Act 1989, s 1(2A).

14 NHS (Reform and Healthcare Professions) Act 2002, s 25(2A).

15 Medical Act 1983, ss 29(4) and 40A. In the case of doctors, the ‘tribunals’ are the Medical Practitioners Tribunals, described in s 2A below.

16 In fact, despite the judiciary’s long-standing role in professional regulation via doctors’ appeals, generating a ‘haystack’ of many hundreds of court judgments, detailed examination of the courts’ role as moderator in the ‘regulatory bargain’ rarely features in academic literature.

17 A Worsley and others, ‘Protecting the Public? An Analysis of Professional Regulation: Comparing Outcomes in Fitness to Practice Proceedings for Social Workers, Nurses and Doctors’ (2020) 50(6) British Journal of Social Work 1871.

18 It is the only other healthcare regulator to have this power: *Gross Negligence Manslaughter in Healthcare: The Report of a Rapid Policy Review* (2018) 11.1 (the Williams Review).

19 Namely *Bolton v Law Society* [1994] 1 WLR 512.

20 Aired in DHSC Consultation, ‘Regulating Healthcare Professions, Protecting the Public’ (2021)—see s 6 below for more detail.

21 Medical Act 1983, s 5.

22 ibid s 2.

23 ibid s 29A.

24 ibid s 35.

25 ibid Part V.

26 Between 300 and 400 cases annually: GMC, *Fitness to Practise Statistics 2022* <www.gmc-uk.org/about/what-we-do-and-why/data-and-research/medical-practice-statistics-and-reports/fitness-to-practise>.

27 At an earlier stage, Case Examiners can apply ‘warnings’ or ‘undertakings’ or determine that the case should not go to tribunal: GMC, *Fitness to Practise Rules 2004*, Part 2(8).

28 Medical Act 1983, s 35D.

29 GMC, *Sanctions Guidance* (2023).

30 However, on the limitations of the individual/system distinction, see K Shojania and M Dixon-Woods, ‘“Bad Apples”: Time to Redefine as a Type of Systems Problem?’ (2013) 22 BMJ Quality & Safety 528.

31 Appointed to oversee the doctor’s revalidation: Medical Practitioner (Responsible Officer) Regulations 2010, SI 2010/2841.

32 Medical Act 1983, s 35C; *Azzam v GMC* [2008] EWHC 2711, [42]–[44].

33 P Case, ‘The Good, the Bad and the Dishonest Doctor: The General Medical Council and the “Redemption Model” of Fitness to Practise’ (2011) 31(4) LS 591; R Kirkham and others, ‘The Procedural Fairness Limitations of Fitness to Practise Hearings: A Case Study into Social Work’ (2019) 39(2) LS 339.

34
*Yeong v GMC* [2009] EWHC 1923, [48] elaborating on types of complaint which may be more (or less) remediable.

35
*Sanusi v GMC* [2018] EWHC 1388, noting that non-attendance was highly prejudicial to the outcome.

36 eg *PSA v GMC & Hanson* [2021] EWHC 588, [26].

37 The importance of insight and remediation is spelled out in many cases, including *PSA v GMC (Battah)* [2022] EWHC 2075.

38 eg *Williams v General Dental Council* [2022] EWHC 1380, [145].

39
*Bramhall* (n 2).

40
*GMC v Sawati* [2022] EWHC 283, [76].

41 Sometimes referenced as a ‘social contract’: M Dixon Woods and others, ‘Why Is UK Medicine no Longer a Self-regulating Profession? The Role of Scandals Involving “Bad Apple” Doctors’ (2011) 73(10) Social Science and Medicine 1452.

42 B Salter, ‘Change in the Governance of Medicine: The Politics of Self-Regulation’ (1999) 27(2) Policy and Politics 143, 144.

43 M Davies, ‘The Future of Medical Self-Regulation in the United Kingdom—Renegotiating the State–Profession Bargain?’ (2014) 14(4) Medical Law International 236, 236.

44 ibid 237.

45 Tracey Adams, ‘Self-regulating Professions: Past, Present, Future’ (2017) 4 Journal of Professions and Organization 70.

46 J Waring, ‘Adaptive Regulation or Governmentality: Patient Safety and the Changing Regulation of Medicine’ (2007) 29(2) Sociology of Health and Illness 163.

47 S Devaney and S Holm, ‘The Transmutation of Deference in Medicine: An Ethico-Legal Perspective’ (2018) 26(2) Med L Rev 202, 219.

48 See cases at nn 91–6.

49 M Davies, *Medical Self-Regulation: Crisis and Change* (Ashgate 2007) 12.

50 Davies, ‘The Future of Medical Self-Regulation’ (n 43) 237.

51 J Chamberlain, *The Sociology of Medical Regulation* (Springer Science 2013) 46.

52 Davies, ‘The Future of Medical Self-Regulation’ (n 43) 236.

53 The General Medical Council (Constitution) (Amendment) Order 2012/1654.

54 MPTs must include a Chair, at least one medical panellist and one lay panellist: *General Medical Council (Constitution of Panels and Investigation Committee) Rules* (2004) rule 4.

55 J Black, ‘Decentring Regulation: Understanding the Role of Regulation and Self-Regulation in a Post-Regulating World’ (2001) 54(1) CLP 103, 104.

56 Davies, ‘The Future of Medical Self-Regulation’ (n 43) 244; A Tazzyman and others, ‘Reforming Regulatory Relationships: The Impact of Medical Revalidation on Doctors, Employers, and the General Medical Council in the United Kingdom’ (2019) Regulation and Governance 593,596.

57 See Devaney and Holm (n 47) 220.

58 M Davies, ‘The Demise of Professional Self-Regulation? Evidence from the “Ideal Type” Professions of Medicine and Law’ (2010) 26(1) Journal of Professional Negligence 3.

59 R Kaye ‘Regulated Self-Regulation: A New Paradigm for Controlling the Professions’ (2006) 21(3) Public Policy & Administration 105.

60 PSA, *Safer Care for All: Solutions from Professional Regulation and Beyond* (2022) 4.

61 O Quick, *Regulating Patient Safety* (CUP 2017) 82.

62
*Sanctions Guidance* (2024) <www.mpts-uk.org/-/media/mpts-documents/07_-dc4198-sanctions-guidance-5-february-2024_pdf-104619554.pdf>.

63 Using ‘section 60 orders’: s 60 of the Health Act 1999 references a fast-track parliamentary approval process (the ‘Order in Council’), providing the GMC and other regulators with great flexibility to change their procedures, with greater rule making flexibility being introduced under Schedule 4 of Anaesthesia Associates and Physician Associates Order 2024, SI 2024/374.

64 See n 53 above.

65 J Waring and others, ‘Modernising Medical Regulation: Where Are We Now?’ (2010) 14(6) Journal of Health Organization and Management 1477.

66 See P Case, ‘Health Care Professions Regulation’ in J Laing and J McHale (eds), *Principles of Medical Law* (5th edn, OUP forthcoming); P Case, ‘Constructing the Good Doctor and the Bad Apple through Insight and Remediation’ (forthcoming).

67
*The Report of the Public Inquiry into Children’s Heart Surgery at the Bristol Royal Infirmary 1994–1995* (2001) (the Bristol Inquiry), as summed up in *CRHP v GMC & Ruscillo* [2004] EWCA Civ 1356, [74].

68 The PSA’s Director of Policy & Standards articulated its function as being ‘to ensure that regulation tilts towards public protection as opposed to professional self-interest’: DHSC, *Report of the Independent Inquiry into the Issues Raised by Paterson* (2020) 190 (Paterson Inquiry).

69 DHSC, *Learning from Tragedy, Keeping Patients Safe* (2001) 2.17.

70 DHSC, *Report of the Independent Inquiry* (n 68) 221.

71 See eg Dame Janet Smith, speaking of her conclusions after leading the Shipman Inquiry: ‘I came to realise that the GMC was capable of saying the right thing … and talking convincingly about patient protection … but, when push came to shove, its members voted in favour of self-protection’: Dame Janet Smith, ‘The Role of the Doctor in the Process of Medical Regulation’ (2006) 12 Clinical Risk 96, 97. See also Devaney and Holm (n 47) 219.

72 eg *GMC v Stone* [2017] EWHC 2534, [58].

73 Medical Act 1983, s 1(1B).

74 ibid.

75 eg *Sanctions Guidance* (n 62) paras 4 and 14–15, under ‘Why do we impose sanctions?’

76 The last 12 months of decisions are accessible via the MPTS site or individual decisions; sometimes older than 12 months can be accessed via the doctor’s record on the online register <www.gmc-uk.org/registration-and-licensing/the-medical-register>.

77 ‘Performativity’ in the sense of processes which bring about a certain kind of reality, eg the repetition of the public protection formula constitutes a shared understanding of what ‘protecting the public’ involves. The many meanings of performativity are often attributed to the work of Judith Butler, eg J Butler, ‘Performative Agency’ (2010) 3(2) Journal of Cultural Economy 147.

78 H Mistry, ‘A Performative Theory of Judicial Dissent’ (2023) 86(3) MLR 729, 755, examining ritual in judicial speech acts.

79 Under Medical Act 1983, s 40.

80 eg *R (on the application of McHugh) ex parte GMC* [2023] NIKB 46—patients challenging the legality of allowing a doctor’s voluntary erasure.

81
*Ruscillo* (n 67) [2], [6].

82 eg through the use of MPTS ‘Circulars’ <www.mpts-uk.org/about/circulars>.

83 Health and Social Care Act 2015, s 5, amending s 25(2) of the NHS Reform and Health Professions Act 2002.

84 Under s 29(4) of the NHS Reform and Healthcare Professions Act 2002.

85 Medical Act 1983.

86 As per common law and art 6 of the European Convention on Human Rights.

87 Medical Act 1983, s 1(3)(g).

88 As a statutory committee of the GMC (ibid).

89 A ‘right’ not a ‘duty’ to appeal: *PSA v GMC & Dighton* [2021] EWHC 32, [20].

90 General Medical Council (Fitness to Practise and Over-arching Objective) and the Professional Standards Authority for Health and Social Care (References to Court) Order 2015, SI 2015/794, para 18. The original version of the PSA’s power in s 29 above focused on ‘undue leniency’.

91
*Evans v GMC* [1984] UKPC 48; *Ghosh v GMC* [2001] 1 WLR 1915.

92
*Marinovich v GMC* [2002] UKPC 36, [28] (Lord Hope). See also the extract below, text accompanying n 148.

93
*Marinovich* (n 92).

94 ‘Decisions on fitness to practise such as assessing the seriousness of any misconduct, may turn on an exercise of professional judgement. In this regard respect must be accorded to a professional disciplinary tribunal’ IGMC v *Cheatle* [2009] EWHC 645, [15].

95
*Evans* (n 91) 3.

96 ibid. The committee was ‘familiar with’ the gradations of seriousness and ‘peculiarly well qualified’ to identify ‘infamous conduct in a professional respect’). This concept was defined by Scrutton LJ in *R v GMC* [1930] 1 KB 562, 569 as ‘serious misconduct *judged by professional standards*’ (author’s emphasis)).

97 Devaney and Holm (n 47) 203.

98 The term ‘reasoned judgment’ is used to distinguish reporting of ‘consent orders’, which are excluded from this study—see eg <www.professionalstandards.org.uk/docs/default-source/section-29/consent-orders/gmc/210817-borse-consent-order.pdf?sfvrsn=97614920_2>.

99 A (then) complete list of GMC appeals was taken largely from the minutes of the Council meeting of 29 September 2022 <www.gmc-uk.org/-/media/documents/council-meeting---29-september-2022---agenda-and-articles-new_pdf-96549629.pdf> and the list of PSA appeals <www.professionalstandards.org.uk/what-we-do/our-work-with-regulators/decisions-about-practitioners/cases-appealed>.

100
*GMC v Jagjivan* [2017] EWHC 1247; see s 4 below.

101 <www.gmc-uk.org/registration-and-licensing/the-medical-register#searchTheRegister>.

102 See the Appendix for a breakdown of the cases by type.

103 The term ‘deference’ has many varied applications: see eg M Elliott, ‘From Bifurcation to Calibration: Twin-Track Deference and the Culture of Justification’ (University of Cambridge 2015) Legal Studies Research Article Series, 9.

104 A Nurse, *The Citizen and the State: Criminal Justice and Civil Liberties in Conflict* (Emerald Publishing 2000) 42.

105 eg G Lawson and G Seidman, *Deference: The Legal Concept and the Legal Practice* (OUP 2019) 80.

106 D Mantzari, ‘Economic Evidence in Regulatory Disputes: Revisiting the Court–Regulatory Agency Relationship in the US and the UK’ (2016) 36(3) OJLS 565.

107 See C Chan, ‘A Preliminary Framework for Measuring Deference in Rights Reasoning’ (2016) 14(4) ICON 851, 853, noting that meaningful studies of deference are almost inevitably qualitative.

108 Elliott (n 103) 10–15.

109 Deference usually being accorded to ‘evaluative judgements’ (see *Bawa-Garba v GMC* [2018] EWHC 76, discussed below), and some public lawyers regarding interference with ‘weighing’ of relevant factors as stretching the boundaries of reasonableness ‘review’, and more consistent with proportionality review (although that is contested): P Craig, ‘The Nature of Reasonableness Review’ (2013) 66(1) CLP 131.

110 Recommending a radical course of treatment whilst knowing it to be inappropriate.

111 Repeated comments and unwanted touching in a ‘hierarchical context’, the nurse being relatively junior.

112
*Sastry v GMC* [2021] EWCA Civ 623, [112]–[113]. Although, after reviewing the tribunal’s decision, the Court of Appeal determined that the tribunal’s orders for erasure were not wrong and should stand.

113 ibid 107–8.

114
*GMC v Bawa-Garba* [2018] EWCA Civ 1879, [67] (*Bawa-Garba* appeal); *Sastry* (n 112) [44].

115 Under s 40 of the Medical Act 1983.

116
*Sastry* (n 112) [105].

117 See eg *GMC v Theodoropoulos* [2017] EWHC 4794, [48]; *GMC v Narayan* [2017] EWHC 2695; *GMC v Somuah-Boateng* [2017] EWHC 3565, [52]–[53], using language of ‘appropriateness’ more consistent with an appeal than a review/appearing to adopt a proportionality standard.

118 T Sayers and CRG Murray, ‘A Tale of Two Doctrines: Revaluating Bifurcation in Substantive Review before the Supreme Court’ [2021] PL 47.

119 Where appeals work asymmetrically, they introduce distortion in regulation, eg like a one-way ratchet or bias. See M Wasserman, ‘Deference Asymmetries: Distortions in the Evolution of Regulatory Law’ [2014–15] Tex L Rev 625.

120 eg *Raychaudhuri v GMC* [2019] EWCA Civ 2027, [57].

121 The PSA’s expertise comes from working with professional regulators, research and an overview of what works and what does not in 10 sectors <www.professionalstandards.org.uk>.

122
*Ghosh* (n 91) 1923G.

123
*Stone* (n 72) [34].

124 As set out in Michael Taggart’s seminal piece, ‘Proportionality, Deference, Wednesbury’ [2008] NZ L Rev 428.

125 Justice Morris in *Kefala v GMC* [2020] EWHC 2480, [13].

126 Two styles of deferential and non-deferential judging were observed by Montgomery in medical law jurisprudence generally: J Montgomery, ‘Law and the Demoralisation of Medicine’ (2006) 26(2) LS 185.

127 ibid 203. See also the cases identified below at n 185.

128 See the cases listed below at n 184.

129
*Jagjivan* (n 100).

130
*Bawa-Garba* appeal (n 114).

131
*Jagjivan* (n 100) [39].

132 A rare exception being *GMC v Awan* [2020] EWHC 1553.

133
*Jagjivan* (n 100) [40] (iii).

134 ibid [40] (iv).

135 ibid [44].

136 ibid [40], (v).

137 ibid [40]. See also *Khan v GMC* [2008] EWHC 3509, [77] (on dishonesty); *Stone* (n 72) [34] and *MXM v GMC* [2022] EWHC 817, [84] (on sexual misconduct).

138
*Jagjivan* (n 100).

139 See eg *GMC v Armstrong* [2021] EWHC 1558, [33] (on dishonesty), where the court was ‘in territory where specialist expertise is less likely to play a decisive role’.

140
*Evans* (n 91) 5.

141
*Ghosh* (n 91) [34].

142
*Bawa-Garba* appeal (n 114).

143 P Case and G Sharma, ‘Promoting Public Confidence in the Medical Profession: Learning from the Case of Dr Bawa-Garba’ (2020) 20(1) Medical Law International 58 <https://doi.org/10.1177/0968533220933788>.

144
*Bawa-Garba* (n 109).

145 ibid [67].

146
*Crabbie v GMC* [2002] UKPC 45, [25]; *Bawa-Garba* (n 109) [67]; *Adil v GMC* [2023] EWCA Civ 1261, [39].

147
*Bramhall* (n 2) [32].

148
*GMC v Mok* [2022] EWHC 1651, [48].

149
*Marinovich* (n 92) [28] (emphasis added). The *Bawa-Garba* judgment observed that this was ‘*particularly* true’ in cases of professional performance (*Bawa-Garba* (n 109) [95]).

150
*Bawa-Garba* (n 109) [67].

151
*GMC v Raychaudhuri* [2018] EWCA Civ 2027, [56]–[57]. This is not an isolated case; see also *Kefala* (n 125).

152
*GMC v Ahmed* [2022] EWHC 403, [67].

153
*Mok* (n 148) [48].

154 See eg *Bramhall* (n 2) [41], where Collins Rice J categorised the case as concerning an evaluative judgment about the seriousness of Bramhall’s conduct in initialling a patient’s liver with a coagulator at the end of a transplant (particularly given the different ways of reading this conduct—violence targeting vulnerable patients *or* silly hubris), but proceeded to apply a very stringent process of review—discussed below at (iii).

155 It is impossible to be sure on causality, ie whether the judge’s framing of the case played a part in determining the approach and outcome or whether the framing was chosen so as to support the judge’s conclusions: Frank B Cross, ‘Political Science and the New Legal Realism’ (1997) 92 Northwestern University Law Review 251, 299.

156 See *Theodoropoulos* (n 117); *Stone* (n 72); *Somuah-Boateng* (n 117); *GMC v Khetyar* [2018] EWHC 813; *GMC v Mmono* [2018] EWHC 3512; *GMC v Zafar* [2020] EWHC 846; *PSA v GMC & Austin* [2022] CSIH 37; *Hanson* (n 36); *PSA v GMC & Dighton* [2020] EWHC 3122; *PSA v GMC & Hilton* [2019] EWHC 1638; *GMC v Rezk* [2023] EWHC 3228.

157 See Chan (n 107) 857, noting that outcomes can be determined by the strength of the applicants’ case.

158 eg *Zafar* (n 156), where the appeal would have succeeded anyway, given findings that new evidence from the criminal courts had not been disclosed to the original tribunal; see also *GMC v Krishnan* [2017] EWHC 2892, where the tribunal had applied the wrong test, the test for dishonesty having been changed by the Supreme Court.

159 Law Commission, *Regulation of Healthcare Professions* (2012) 201. For another instance of the GMC underlining its separateness from MPTS decision making, see its very public disagreement with the MPTS decision to allow the voluntary erasure of Dr Watt: [2023] NIKB 46, [31].

160 See the text accompanying nn 75–8.

161 The PSA was sceptical of this as offering ‘an appearance of separation … without substance’: *Response to the consultation ‘General Medical Council and Professional Standards Authority: Proposed changes to modernise and reform the adjudication of fitness to practise cases* (PSA, September 2014) 1.8; and it ‘will be both the appellant and the primary respondent … in the cases it refers’ (5.2).

162 See *Jagjivan* (n 100); see also the text accompanying nn 133–5.

163 On dishonesty, see *Bolton v Law Society* [1994] 1 WLR 512; on sexual probity see *GMC v Chandra* [2018] EWCA Civ 1898.

164 See eg *Hilton* (n 156).

165 eg *Rezk* (n 156).

166 It is widely known that doctors’ appeals under s.40 of the Medical Act 1983 rarely succeed: https://doctorsdefenceservice.com/appeals-from-gmc-decisions/.

167 M Galanter, ‘Why the “Haves” Come Out Ahead: Speculations on the Limits of Legal Change’ (1974) 9(1) L & Soc’y Rev 95, 98.

168 See Chan (n 107) 853.

169 Elliott (n103) 9.

170
*Phipps v GMC* [2006] EWCA Civ 397, [106].

171
*Sanctions Guidance* (n 62) paras 55, 109, 149 and 150.

172
*GMC v MPTS (Mehta)* [2018] CSIH 69, 13.

173
*Awan* (n 132) [12].

174 These include: causing serious harm to others; sexual offences; dishonesty; abuse of position/trust,;putting own interests above those of the patient; and a persistent lack of insight.

175 Discussed below in s 5A(ii).

176
*GMC v Donadio* [2021] EWHC 562, [71].

177 P Case, ‘Doctoring Confidence and Soliciting Trust: Models of Professional Discipline in Law and Medicine’ (2013) 29(2) Journal of Professional Negligence 87.

178
*Donadio* (n 176) [107] (emphasis added).

179 ibid [37].

180 ibid; *Khetyar* (n 156); *Mmono* (n 156); *Bramhall* (n 2); *GMC v Saeed* [2020] EWHC 83. *Battah* (n 37) [63] is probably also an example.

181 It is worth noting that in *Bawa-Garba* (n 108) [40], Ouseley J (approved in *Bawa-Garba* appeal (n 113)) had rejected the suggestion ‘that (in that case) a conviction for gross negligence manslaughter gave rise to a presumption of erasure’; that was not the wording of the *Sanctions Guidance* (n 62).

182
*Khetyar* (n 156) [20].

183
*Bramhall* (n 2) [38] (author’s emphasis).

184 See eg J Goodwin, ‘The Last Defence of Wednesbury’ [2012] PL 445.

185 In some instances, the court appeared to blur review standards, adopting a broader threshold of ‘appropriateness’: see eg *Theodoropoulos* (n 117) [48] (erasure was the ‘appropriate’ sanction for dishonesty which went to the integrity of the regulatory system); *Mmono* (n 156) [28]. In others, the court dwells on disagreement, with the ‘weight’ attached to a relevant consideration or interference with a balancing judgment: *Nwachuku* [2017] EWHC 2875, [56] (too much weight given to doctor’s reflective statement and (late) admission of dishonesty and testimonials); *Chaudhury* [2017] EWHC 2561, [50] (incorrect balancing); *Stone* (n 72) [55] and [58] (excessive weight given to testimony of psychologist, so that the tribunal had not truly grappled with seriousness); *Chandra* (n 163) [50]; *GMC v Patel* [2018] EWHC 171, [60] (too much weight given to remediation); *Somuah-Boateng* [2018] EWHC 3565 (court seemed to know better on the evaluative issue of public confidence); *GMC v Nyamasve* [2018] EWHC 1689, [39] (insufficient weight had been given to the doctor’s dishonesty); *GMC v Haris* [2020] EWHC 2518, [65] (the court substituted its own view on sexual motive); *Armstrong* (n 139) [53] and [55] (intervention was on the basis of what constitutes an ‘exceptional case’ and too much weight being placed on mitigation); *Bramhall* (n 2) [39] (categorised as an evaluative judgment, but then still does interfere with it, including because too much weight was attached to mitigation).

186 See the unsuccessful appeals of *Raychaudhri* (n 151) (although concerning dishonesty, framed as an evaluative/multifactorial decision which tribunal best placed to make); *Bawa-Garba* appeal (n 114) (see particularly [67] on the approach to *Sanctions Guidance* (n 62)); *GMC v X* [2019] EWHC 493 (relying on *Bawa-Garba* and its approach to evaluative decisions, even though this was sexual misconduct and dishonesty, and the non-binding nature of the *Sanctions Guidance* [68]–[69]); *Awan* (n 132) (note Mostyn J’s preamble, which seems to set the decision up as one going down the path of deference ([8]–[13]), treating sexual misconduct as an evaluative decision, relying on *Bawa-Garba* and not citing *Jagjivan* at all, and referencing the *Sanctions Guidance* as ‘signposts’ and not a ‘fixed track’ ([11])); *Ahmed* (n 152) (emphasising the non-prescriptiveness of the *Sanctions Guidance* [85]–[87], clearly giving the tribunal the benefit of the doubt where reasoning had not been explicit [98]–[102]); *Mok* (n 148) (treating a sexual misconduct case as coming down to an evaluative decision about sanction [63] and again excusing the tribunal for not being explicit in some of its reasoning [66]).

187
*Chandra* (n 163) [79].

188
*Hanson* (n 36).

189
*PSA v GMC & Onyekpe* [2023] EWHC 2391 (MPT’s finding insufficient due to a failure to explore patient’s vulnerability, as disclosed in her WhatsApp messages, and the doctor’s awareness of it [105]).

190
*Somuah Boateng* (n 117).

191
*Haris* (n 185) [59].

192 Lord Woolf, ‘Are Courts Excessively Deferential to the Medical Profession?’ (2001) 9 Med L Rev 1.

193
*PSA v GMC & Lingam* [2023] EWHC 967, [73]; *Zafar* [87]; *Bramhall* (n 2) [31].

194
*PSA v GMC & Austin* [2023] CSIH 87, [23]; *Battah* (n 37) [39]; *PSA v GMC & Igwilo* [2016] EWHC 254, [29].

195 Appearing throughout *Donadio* (n 176).

196 ibid.

197 Following and elaborating upon *Nyamasve* (n 185) and followed in *Battah* (n 37) and *Austin* (n 156).

198
*Donadio* (n 176) [46].

199
*Donadio* went even further, defining a failure to comply with regulatory requirements as being of grave import in and of itself, independently of any dishonesty: ibid [68].

200
*Mmono* (n 156) [28].

201
*Nyamasve* (n 185) [33] (working as a locum despite not having completed steps to regain his relinquished licence to practise).

202
*Udoye* [2021] EWHC 1511, [65]–[66].

203
*Zafar* (n 156) (dishonesty); *Haris* (n 185) (sexual misconduct); *Armstrong* (n 139) [51] (dishonesty); *Donadio* (n 176) [65] (dishonesty); *Bramhall* (n 2) [59] (assault/criminal conviction).

204
*Sanctions Guidance* (n 61) para 24.

205
*Hilton* (n 156) [117].

206
*Council for the Regulation of Healthcare Professionals v GMC & Southall* [2005] EWCA Civ 579 (Admin), [14] (Collins J). See also Lord Hoffmann in *Bijl v General Medical Council* [2002] Lloyds Rep Med 60, 62.

207
*Giele v GMC* [2005] EWHC 2143, [29]; see also the case of Justin Stebbings, who seemingly escaped erasure in 2021 partly due to 1000 pages of testimonials <www.standard.co.uk/news/london/justin-stebbing-london-cancer-doctor-harley-street-suspended-misconduct-b973007.html>; J Wellesley and E Tumilty, ‘“Saviour or Sinner?” Why the Case of Justin Stebbing Matters’ (*BMJ Journal of Medical Ethics blog*) <https://blogs.bmj.com/medical-ethics/2022/11/13/saviour-or-sinner-why-the-case-of-justin-stebbing-matters/>.

208
*Chaudhury* (n 185) [29].

209 eg as per *Nyamasve* (n 185) [24].

210
*Nwachuku* [2017] EWHC 2085, [55]; *Bramhall* (n 2) [39]—a reasonable reader may have thought that too much weight had been given to testimonials.

211
*Somuah Boateng* (n 117) [53].

212
*Nwachuku* (n 210) [55].

213 On ‘club culture’, see J Chamberlain ‘Regulating the Medical Profession: From Club Governance to Stakeholder Regulation’ (2010) 4(12) Sociology Compass 1035.

214 Many patients are swayed by their doctor’s reputation: Paterson Inquiry (n 68) 218, noting that patients had no means of independently verifying a doctor’s reputation.

215 12 months is the maximum suspension that can be ordered. A review is not automatic, but is now imposed almost by default following suspension: Medical Act 1983, s 35D. As an exception, see *Honey Rose* [2021] EWHC 2888.

216
*Theodoropoulos* (n 117); *Stone* (n 72); *Somuah Boateng* (n 117); *Khetyar* (n 156); *Mmono* (n 156); *Zafar* (n 156); *Austin* (n 156).

217 See s 2A above.

218 Doctors may apply for ‘restoration’ to the register, but not until five years has passed from the order for their erasure: General Medical Council (Voluntary Erasure and Restoration following Voluntary Erasure) Regulations 2004, SI 2004/2609.

219 Further examples include *Austin* (n 156) [25] (suspension being used to ‘test’ whether insight and remediation could be achieved); *Mmono* (n 156).

220 cf *Raschid v General Medical Council* [2007] EWCA Civ 46, [22], where a very trusting approach is evident.

221 N Lacey and H Pickard, ‘To Blame or to Forgive? Reconciling Punishment and Forgiveness in Criminal Justice’ (2015) 35(4) OJLS 665.

222 Case, ‘The Good, the Bad and the Dishonest Doctor’ (n 33) 591.

223
*Somuah Boateng* (n 117) [57]. In another sexual misconduct case, the court referred to the distinction between suspension and erasure as ‘real and substantial’: *Khetyar* (n 156) [18].

224 If the review panel finds that the doctor’s insight and remediation have not improved sufficiently, it may extend the suspension or substitute with erasure: eg as per *Sledzik* (n 1).

225
*Bolton* (n 19) 518–19. For exploration of its use in medical professional regulation on the issue of dishonesty, see n 177 above.

226 Similar terms are repeated in the *Sanctions Guidance* (n 62) para 17: ‘Although the tribunal should make sure the sanction it imposes is appropriate and proportionate, the reputation of the profession as a whole is more important than the interests of any individual doctor.’

227
*Donadio* (n 176); *Chandra* (n 163); *Zafar* (n 156); *Armstrong* (n 139) 51; *Patel* (n 185) [74] (failure to follow the guidance in *Bolton*). *Bolton* is also sometimes cited with approval but without further comment, eg *Nyamasve* (n 185); *Awan* (n 132). In a similar vein, see also the use of *Bolton* in *Hilton* (n 156).

228
*Zafar* (n 156).

229 ibid [83].

230
*Chandra* (n 163).

231
*Patel* (n 185) [64].

232
*Chandra* (n 163) generally and at [92]; *Patel* (n 185) [63]; *Zafar* (n 156); *Donadio* (n 176) [65].

233 eg *Singh v GMC* [2000] UKPC 15; *Bawa-Garba* (n 109) [10].

234
*Bolton* (n 19) [16].

235 The wording does therefore arguably catch what is said above about positive testimonials from peers (and as noted above, there is scope for real concern about placing too much weight on positive testimonials).

236 Discussed in s 2A above.

237
*Sanctions Guidance* (n 62) para 25.

238 See *The Just Culture Guide* (NHS Resolution 2023); S Dekker, *Just Culture: Balancing Safety and Accountability* (CRC Press 2012); V Moore, ‘Doctors, Decisions, and Discharges: Regulatory Accountability for Patient Safety in a Just Culture’ (2020) 36(4) Journal of Professional Negligence 171.

239 For one of many examples, see *Sawati* (n 40) [76], linking insight with future risk.

240 On candour in healthcare and policy and legal developments on this front, see O Quick, ‘Duties of Candour in Healthcare: The Truth, the Whole Truth and Nothing but the Truth’ (2022) 30(2) Med L Rev 324.

241 eg Professional Standards Authority, How Is Public Confidence Maintained When Fitness to Practise Decisions Are Made? (2019) 1.6, describing it as ‘out of step’.

242 See s 2B above and text accompanying nn 71–8.

243
*Bolton* (n 19) [15].

244 ibid.

245 See eg Professional Standards Authority, ‘Dishonest Behaviour by Health and Care Professionals’ (2016), noting the more nuanced and tolerant approach to disposal of cases of dishonesty the public appeared to have (except for the most egregious cases), and both professions and the public reportedly being relatively sceptical of claimed threats to public confidence where patient safety was not clearly in issue.

246 Medical Act 1983, s 35C; see also n 32 above—‘impairment’ does not feature in solicitors’ regulation in the UK.

247 See Case, ‘The Good, the Bad and the Dishonest Doctor’ (n 33), noting a distinct emphasis on redemption in the medical model of fitness to practise, unlike in the legal profession.

248 ‘The decision whether to strike off or to suspend will often involve a fine and difficult exercise of judgement, to be made by the Tribunal as an informed and expert body on all the facts of the case’: *Bolton* (n 19) [14].

249 ibid [15].

250 Set out in the text accompanying nn 34–40.

251
*Bolton* (n 19) [15].

252
*Bramhall* (n 2) being an exception. The workings and merits of this redemptive approach are explored in a separate article: P Case, ‘Professional Regulation and Attitudinal Issues: Constructing the Good Doctor and the Bad Apple through Insight and Remediation’ (forthcoming).

253 eg *Sledzik* (n 1); *GMC v Walton* [2019] EWHC 3537—the GMC’s appeals being successful, but the court clearly not agreeing with much of the GMC’s argument and adopting a deferential tone.

254
*Donadio* (n 176), following and elaborating upon *Nyamasve* (n 185) and followed in *Battah* (n 37); *Austin* (n 156).

255
*GMC v Armstrong* [2021] EWHC 1658, [33].

256 The government committed to removing s 40A in 2018, but changes have not yet been introduced: E Mahase, ‘Nineteen Healthcare Organisations Urge Government to Strip GMC of Its Power to Appeal’ (2023) BMJ 1328.

257 The first phase of enactment is now transposed into the Anaesthesia Associates and Physician Associates Order 2024 (n 63), but rollout to doctors is still in the planning phases.

258 ibid art 10, particularly 10(8), which enables case examiners to impose sanctions (‘final measures’) where the examiner finds fitness to practise impaired and the registrant accepts the examiner’s findings.

259 P Sanderson, *From Public Hearings to Consensual Disposal: Insights from the Literature* (PSA 2019).

260 ‘[T]he MPT have not had days of evidence including cross-examination in which to acquaint themselves fully with the nuance of the behaviour which is under examination’: *Lingam* (n 193) [76].

261 ibid [73] and [79].

262 Although they are discussed further in Case, ‘Health Care Professions Regulation’ (n 66) paras 2.52–2.53.

## *Appendix* Reasoned Judgments in Public Protection Appeals Concerning Doctors: 2017–Date

**Table:**

	Case	Allegation type
1.	*GMC v Jagjivan* [2017] EWHC 1247	Sexual misconduct + dishonesty
2.	*GMC v Theorodopoulos* [2017] EWHC 4794	Dishonesty
3.	*Nwachuku v GMC* [2017] EWHC 2085	Dishonesty
4.	*GMC v Raychaudhuri* [2018] EWCA Civ 2027	Dishonesty
5.	*GMC v Narayan* [2017] EWHC 2695	Sexual misconduct
6.	*GMC v Chaudhary* [2017] EWHC 2561	Dishonesty
7.	*GMC v Stone* [2017] EWHC 2534	Sexual misconduct
8.	*GMC v Chandra* [2018] EWCA Civ 1898	Sexual misconduct
9.	*GMC v Krishnan* [2017] EWHC 2892	Dishonesty
10.	*GMC v Lamming* [2017] EWHC 3309	Dishonesty
11.	*GMC v Nooh* [2017] EWHC 2948	Clinical competence/Dishonesty
12.	*GMC v Patel* [2018] EWHC 171	Dishonesty
13.	*GMC v Somuah-Boateng* [2017] EWHC 3565	Sexual misconduct
14.	*GMC v Bawa-Garba* [2018] EWCA Civ 1879	Criminal conviction
15.	*GMC v Khetyar* [2018] EWHC 813	Sexual misconduct
16.	*GMC v Nyamasve* [2018] EWHC 1689	Dishonesty
17.	*GMC v MPTS (Mehta)* [2018] CSIH 69	Sexual misconduct
18.	*GMC v Mmono* [2018] EWHC 3512	Sexual misconduct/Dishonesty
19.	*PSA v GMC & Hilton* [2019] EWHC 1638	Dishonesty
20.	*GMC v Sledzik* [2019] EWHC 189	Clinical competence
21.	*GMC v X* [2019] EWHC 493	Sexual misconduct
22.	*GMC v Zafar* [2020] EWHC 846	Dishonesty
23.	*GMC v Saeed* [2020] EWHC 83	Criminal conviction
24.	*GMC v Walton* [2019] EWHC 3537	Dishonesty
25.	*GMC v Haris* [2020] EWHC 2518	Sexual misconduct
26.	*GMC v Awan* [2020] EWHC 1553	Sexual misconduct
27.	*PSA v GMC & Dighton* [2020] EWHC 3122 and then [2021] EWHC 32	Clinical competence/Persistent lack of insight
28.	*GMC v Armstrong* [2021] EWHC 1558	Dishonesty
29.	*GMC v Udoye* [2021] EWHC 1511	Dishonesty
30.	*GMC v Donadio* [2021] EWHC 562	Clinical competence/Dishonesty
31.	*PSA v GMC & Hanson* [2021] EWHC 588 and then [2021] EWHC 1288	Sexual misconduct
32.	*GMC v Ahmed* [2022] EWHC 403	Sexual misconduct
33.	*GMC & PSA v Bramhall* [2021] EWHC 2109	Criminal conviction
34.	*PSA v GMC & Austin* [2022] CSIH 37	Dishonesty
35.	*PSA v GMC & Battah* [2022] EWHC 2075	Clinical competence/Possible dishonesty
36.	*GMC v Mok* [2022] EWHC 1651	Sexual misconduct
37.	*PSA v GMC & Lingam* [2023] EWHC 967	Clinical competence
38.	*PSA v GMC & Onyekpe* [2023] EWHC 2391	Sexual misconduct
39.	*GMC v Rezk* [2023] EWHC 3228	Sexual misconduct
40.	*GMC v Dugboyele* [2024] EWHC 2651	Sexual misconduct

